# Optimal Oral Antithrombotic Regimes for Patients with Acute Coronary Syndrome: A Network Meta-Analysis

**DOI:** 10.1371/journal.pone.0090986

**Published:** 2014-03-10

**Authors:** Yicong Ye, Hongzhi Xie, Yong Zeng, Xiliang Zhao, Zhuang Tian, Shuyang Zhang

**Affiliations:** Department of Cardiology, Peking Union Medical College Hospital, Peking Union Medical College and Chinese Academy of Medical Sciences, Beijing, China; Universidad Peruana de Ciencias Aplicadas (UPC), Peru

## Abstract

**Objective:**

We performed a network meta-analysis to investigate the optimal antithrombotic regime by indirectly comparing new antithrombotic regimes (new P2Y12 inhibitors plus aspirin or novel oral anticoagulants on top of traditional dual antiplatelet therapy [DAPT]) in patients with acute coronary syndrome (ACS).

**Methods:**

A systematic search of MEDLINE, EMBASE, and the Cochrane databases was performed to identify all phase 3 randomized controlled trials (RCTs) involving novel oral anticoagulants or oral P2Y_12_ inhibitors in patients with ACS. Major adverse cardiac events (MACE) were regarded as the efficacy endpoint, and thrombolysis in myocardial infarction (TIMI) major bleeding events were used as the safety endpoint. The net clinical benefit was calculated as the sum of MACE and TIMI major bleeding events.

**Results:**

Five phase 3 RCTs with 64,476 ACS patients were included. Although there were no significant differences among new antithrombotic regimes, rivaroxaban 5 mg twice daily plus traditional DAPT might be the most effective in reducing the incidence of MACE, accompanying the highest risk of TIMI major bleeding. Ticagrelor plus aspirin presented slight advantage on the net clinical benefit over other new antithrombotic regimes, with the highest probability of being the best regimes for net clinical benefit (35.0%), followed by prasugrel plus aspirin (28.0%), and rivaroxaban 2.5 mg twice daily plus traditional DAPT (19.5%).

**Conclusion:**

Novel antithrombotic regime with ticagrelor plus aspirin brings a larger clinical benefit in comparison with other regimes, suggesting that it may be the optimal antithrombotic regime for patients with ACS.

## Introduction

It is well known that the formation of thrombosis is the major pathophysiologic mechanism of acute coronary syndrome (ACS), and thus traditional dual antiplatelet therapy (DAPT) (aspirin in combination with thienopyridines, predominantly clopidogrel) has become the mainstay of treatment in patients with ACS. Nevertheless, there remains about 10% risk of recurrent thrombotic events within one year after percutaneous coronary intervention (PCI), even after the use of traditional DAPT [1].

Recently, more intensive antithrombotic regimes have been developed in order to overcome this issue, and the safety and efficacy of these therapies have been verified by a series of randomized clinical trials (RCTs). Newly developed antiplatelet agents (P2Y_12_ receptor inhibitors, e.g. Cangrelor [intravenous], Elinogrel [intravenous], prasugrel [oral] and ticagrelor [oral]) have been shown to have more potent therapeutic effect and have faster onset of action, as well as significantly decrease cardiovascular mortality after PCI as compared to clopidogrel [2]. These advantages make P2Y12 inhibitors particularly attractive to patients with ACS. On the other hand, novel oral anticoagulants, such as rivaroxaban, apixaban, darexaban and dabigatran, have also been developed. A recent meta-analysis in ACS patients has demonstrated that use of the novel oral anticoagulant agents, on top of single antiplatelet regimens, or DAPT in ACS is associated with 30% reduction in recurrent ischemic events, but a substantial increase in bleeding, which is most pronounced when novel oral anticoagulants are prescribed in addition to DAPT [3].

Based on the above clinical evidence, new antithrombotic agents, in addition to DAPT, have been recommended in specific subsets among ACS patients in the current clinical practice guidelines [4]. However, to date there is not any large scale head-to-head trial to compare the clinical utility of these new antithrombotic agents. It is also unclear whether the new DAPT using ticagrelor or prasugrel has superiority to novel oral anticoagulants on top of traditional DAPT in ACS subjects.

We therefore conducted a network meta-analysis based on the available data from published RCTs to investigate the efficacy and safety of these new antithrombotic agents in patients with ACS.

## Methods

### Data Sources and Searches

We conducted a computerized literature search of MEDLINE (1950 to April 2013), EMBASE (1966 to April 2013), and the Cochrane Central Register of Controlled Trials (until April 2013) to identify the eligible studies. An extensive manual search of the literature using the references of the original manuscripts, reviews, and meta-analyses was performed. No language restrictions were imposed. The search strategy was presented in *Text S1*.

### Selection criteria

The clinical trials were eligible for inclusion if 1) study design (phase 3 RCTs) involved patient randomization; 2) participants were diagnosed with ACS; and 3) comparisons were made between new oral P2Y12 receptor inhibitors with clopidogrel and novel anticoagulants with placebo in addition to DAPT. Trials would be excluded if the control group used single antiplatelet treatment, or if the sample size was less than 500.

### Data Extraction

Two authors (Y. Y. and H. X.) independently determined the study eligibility and extracted the following data from the included studies: (1) study design; (2) participant and intervention characteristics; (3) treatment (including novel oral anticoagulants or new oral P2Y12 inhibitors); and (4) clinical outcomes (including major adverse cardiac events [MACE] and Thrombolysis in Myocardial Infarction [TIMI] major bleeding). MACE was defined as a composite endpoint of cardiovascular death, myocardial infarction, or stroke. The net clinical benefit was calculated as the sum of MACE and TIMI major bleeding events. Any disagreements were resolved by consensus, and the principal investigators resolved any disagreements.

### Risk of Bias Assessment

The internal validity of the eligible studies was assessed according to the Cochrane Collaboration risk of bias tool [5]. The risk of bias was described and assessed in seven specific domains: 1) random sequence generation; 2) allocation concealment; 3) blinding of participants, personnel; 4) blinding of outcome assessment; 5) incomplete outcome data; 6) selective reporting; and 7) other sources of bias. The judgments involved using the answers “yes” (indicating a low risk of bias), “no” (indicating a high risk of bias), and “unclear” (if risk of bias is unknown, or if an entry is not relevant to the study).

### Data Synthesis and Analysis

The κ statistic was used to assess the agreement between reviewers for study selection. In the pair-wise meta-analysis, the pooled odd ratio (OR) was calculated for each outcome using the Mantel-Haenszel method for random effects [6]. The heterogeneity across the included studies was assessed using the Cochrane Q test via a χ^2^ test and was quantified with the I^2^ test [7].

A network meta-analysis was conducted to compare the efficacy and safety among these new antithrombotic regimes. Due to indirect comparison among these antithrombotic regimes, it was not feasible to use an inconsistency model or a node-splitting model to statistically identify inconsistencies. We assumed the included studies were consistent based on the criteria of study selection and analysis using a consistency random effects model. This model was implemented in the Bayesian framework and estimated using Markov chain Monte Carlo (MCMC) methods [8], which was recommended by the National Institute for Health and Clinical Excellence (NICE) Decision Support Unit technical support documentson evidence synthesis [9]. The models were run for 300,000 iterations, after which convergence was assessed using the Brooks-Gelman-Rubin diagnostic [9]. Specifically, convergence was assessed by comparing within-chain and between-chain variance to calculate the Potential Scale Reduction Factor (PSRF) [10]. If the PSRF is large, it means that the between-chains variance can be decreased by running additional iterations. If the PSRF is close to 1, it indicates approximate convergence has been reached.

Sensitivity analysis, including both phase 2 and phase 3 studies, was conducted to test how robust the final ranking of these new antithrombotic regimes was relative to eligibility criteria. Publication bias was assessed by the Begg's funnel plot and the Egger weighted regression statistic where a value of *p*<0.10 indicates a significant publication bias among the included studies.

All analyses were performed using STATA (version 11.0) and ADDIS (AggregateData Drug Information System,version 1.16.3). The meta-analysis was prepared in accordance with the PRISMA (Preferred Reporting Items for Systematic Reviews and Meta-Analyses) statement [11].

## Results

### Characteristics of included studies

**Figure 1:**
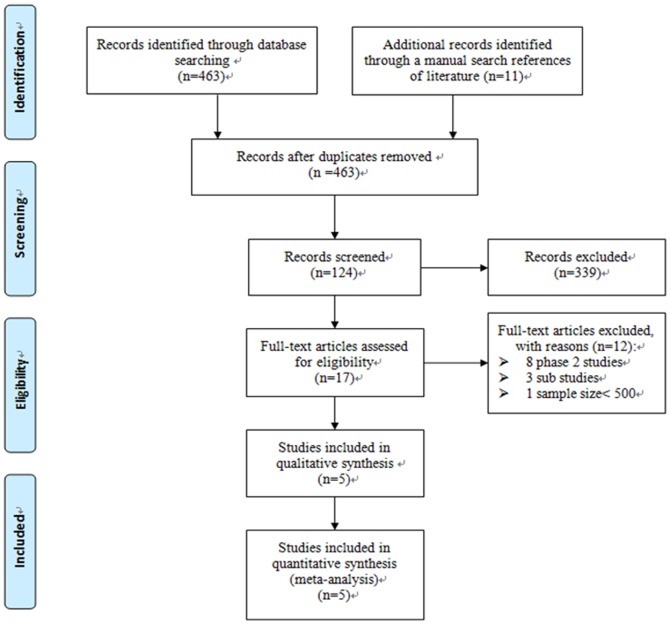
PRISMA flow diagram of study selection.

**Figure 2:**
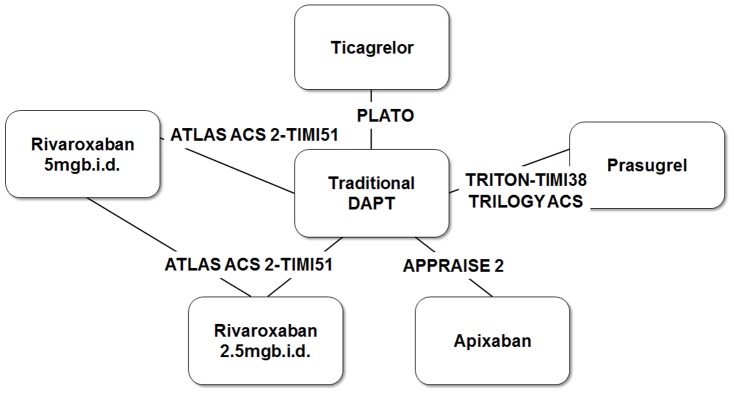
Studies and treatments included in the network meta-analysis.

Four-hundred and sixty-three records were retrieved from the initial search. Seventeen studies were reviewed in full-text. Five phase 3 randomized control trials (TRITON-TIMI 38 [12], TRILOGY ACS [13], PLATO [14], APPRAISE 2 [15], and ATLAS ACS2-TIMI 51 [16]) comparing 5 new antithrombotic regimes with traditional DAPT were included in the meta-analysis (Figure 1 and Figure 2). The inter-reviewer agreement for the study selection was high (κ = 0.98).

Basically, all of the studies included ACS patients with moderate-to-high risk, although the reporting detail of criteria in each study is different (Table S1). The baseline characteristics of the study population were presented in Table 1 and Table S2. A total of 64,476 ACS patients were included in the 5 trials. Of them, 34,864 were randomized to receive new antithrombotic regimes and 29,612 to receive traditional DAPT. The mean or median age of the enrolled patients ranged from 61 to 67 years, and each trial predominantly enrolled men. The median follow-up periods ranged from 8 to 17.1 months. The TRITON-TIMI 38 [12] and TRILOGY ACS trials [13] compared prasugrel plus aspirin with traditional DAPT (clopidogrel plus aspirin) in ACS patients undergoing PCI and non-ST segment elevation ACS patients without PCI, respectively, while the PLATO trial [14] compared ticagrelor plus aspirin with traditional DAPT (clopidogrel plus aspirin) in ACS patients. In APPRAISE 2 [15] and ATLAS ACS2-TIMI 51 trials [16], apixaban and rivaroxaban (two dose regimes: 2.5 mg and 5 mg twice daily) plus traditional DAPT were compared with traditional DAPT (predominately clopidogrel plus aspirin), respectively.

**Table 1:** Characteristics of included studies.

								Active group	Control group
Studies	Year	Study population	Sample size	Age yr	Male%	STEMI%*	Duration Months	New P2Y_12_ inhibitors or novel oral anticoagulants	Thienopyridine %	ASA%	Thienopyridine %	ASA %
TRITON -TIMI38	2007	ACS with PCI	13,608	61	74	26	14.5	Prasugrel 60 mg(LD)+10 mg daily	0	99	100	99
PLATO	2009	ACS	18,624	62	71.7	37.7	9.2	Ticagrelor 180 mg(LD)+90 mg b.i.d	0	97.4	82.8	97.5
APPRAISE 2	2011	ACS	7392	67	67.8	39.6	8	Apixaban 5 mg b.i.d	81	97	81	97
ATLAS ACS2-TIMI 51	2012	ACS	15526	61.7	74.7	50.3	13	Rivoraxaban 2.5/5.0 mg b.i.d	98.7	98.6	92.9	98.7
TRILOGY ACS	2012	NSTEACS without PCI	9326	66	60.8	0	17.1	Prasugrel 30 mg(LD)+5/10 mg daily	0	94	100	93.4

ACS  =  acute coronary syndrome; ASA  =  aspirin; LD  =  loading dose; NSTEACS  =  Non-ST segment elevation acute coronary syndrome; PCI  =  percutaneous coronary intervention; STEMI  =  ST elevation myocardial infarction

### Risk of bias assessment

All trials were high quality multiple-center RCTs with pre-specified protocols, making them low risk of reporting bias (Figure S1).All trials used center randomization and double-blinded methods, and thus had low risk of selection bias and performance bias. The primary and secondary outcomes in all studies were adjudicated with the use of pre-specified criteria by an independent clinical events committee and therefore had a low risk of detection bias. In these included studies, the proportion of missing outcomes was not enough to have a clinically relevant impact on the intervention effect estimate, or missing outcome data balanced in numbers across intervention groups with similar reasons.

### Pair-wise meta-analysis

**Figure 3:**
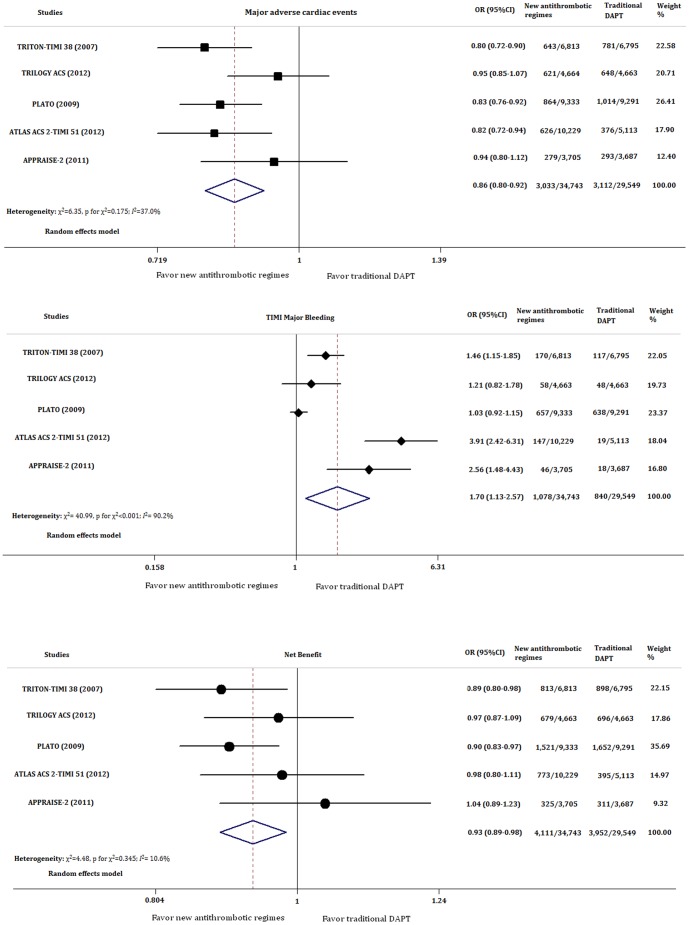
Forest plots of comparisons between new antithrombotic therapy and traditional DAPT in major cardiac adverse events, TIMI major bleeding events and net clinical benefit. DAPT: dual antiplatelet therapy; OR: odd ratio; CI: confidence interval; TIMI: thrombolysis in myocardial infarction.

The use of new antithrombotic agents resulted in significantly reduced risk of MACE compared with traditional DAPT (OR = 0.860; 95% CI, 0.803 to 0.921; p<0.001) with modest heterogeneity (χ^2^ = 6.35, p for χ^2^ = 0.175; *I* ^2^ = 37.0%). However, an increased risk of TIMI major bleeding was identified in new antithrombotic treatment group (OR1.702; 95% CI, 1.125–2.573; *p* = 0.012) with significant heterogeneity (χ^2^ = 40.99, p for χ^2^<0.001; *I* ^2^ = 90.2%). To take the benefit and the bleeding risk together, we found that the use of new antithrombotic agents was associated with a net benefit compared with traditional DAPT (OR = 0.934; 95% CI, 0.888 to 0.983; *p* = 0.009). No significant heterogeneity was identified across the included studies (χ^2^ = 4.48, p for χ^2^ = 0.345; *I* ^2^ = 10.6%; Figure 3)

### Network meta-analysis

In the consistency model, convergence of the model was achieved by extending the number of iterations to 100,000. The ORs and the 95% CIs for all treatments relative to each other under the consistency model were presented in Table 2. Rivaroxaban, either at the dose of 5 mg or 2.5 mg twice daily, presented a beneficial effect of reducing the risk of MACE compared with other agents. However, rivaroxaban was associated with the higher risk of TIMI major bleeding events, especially at the dose of 5 mg twice daily. In addition, ticagrelor seemed likely to have a greater net benefit compared with other antithrombotic agents.

**Table 2:** The odd ratios and the 95% confidence interval for all treatments relative to each other under the consistency model.

Odd ratios and 95% confidential interval for major adverse cardiac events (MACE)				
**Apixaban**	0.92 (0.63, 1.34)	0.87 (0.55, 1.35)	0.87 (0.56, 1.34)	0.88 (0.57, 1.35)
1.08 (0.74, 1.58)	**Prasugrel**	0.94 (0.64, 1.38)	0.94 (0.65, 1.37)	0.96 (0.67, 1.37)
1.15 (0.74, 1.81)	1.06 (0.72, 1.55)	**Rivaroxaban 2.5 mg b.i.d**	1.00 (0.73, 1.37)	1.01 (0.65, 1.56)
1.15 (0.75, 1.79)	1.06 (0.73, 1.55)	1.00 (0.73, 1.37)	**Rivaroxaban 5 mg b.i.d**	1.02 (0.67, 1.55)
1.13 (0.74, 1.74)	1.05 (0.73, 1.50)	0.99 (0.64, 1.53)	0.98 (0.64, 1.50)	**Ticagrelor**

Odd ratios and 95% confidential interval for TIMI major bleeding				
**Apixaban**	0.52 (0.09, 3.22)	1.35 (0.16, 11.05)	1.67 (0.20, 14.10)	0.40 (0.05, 3.33)
1.93 (0.31, 11.15)	**Prasugrel**	2.58 (0.41, 15.56)	3.24 (0.54, 18.99)	0.75 (0.13, 4.45)
0.74 (0.09, 6.21)	0.39 (0.06, 2.41)	**Rivaroxaban 2.5 mg b.i.d**	1.26 (0.29, 5.41)	0.30 (0.04, 2.38)
0.60 (0.07, 4.96)	0.31 (0.05, 1.86)	0.79 (0.18, 3.46)	**Rivaroxaban 5 mg b.i.d**	0.24 (0.03, 1.94)
2.52 (0.30, 19.94)	1.33 (0.22, 7.54)	3.36 (0.42, 25.52)	4.25 (0.52, 33.63)	**Ticagrelor**

Odd ratios and 95% confidential interval for net benefit				
**Apixaban**	0.89 (0.70, 1.13)	0.91 (0.68, 1.22)	0.96 (0.72, 1.28)	0.87 (0.67, 1.13)
1.13 (0.88, 1.43)	**Prasugrel**	1.03 (0.81, 1.29)	1.08 (0.85, 1.36)	0.97 (0.80, 1.19)
1.10 (0.82, 1.46)	0.97 (0.77, 1.23)	**Rivaroxaban 2.5 mg b.i.d**	1.05 (0.86, 1.28)	0.95 (0.74, 1.22)
1.05 (0.78, 1.40)	0.93 (0.74, 1.18)	0.95 (0.78, 1.16)	**Rivaroxaban 5 mg b.i.d**	0.91 (0.70, 1.17)
1.16 (0.89, 1.50)	1.03 (0.84, 1.25)	1.05 (0.82, 1.35)	1.10 (0.86, 1.42)	**Ticagrelor**

**Figure 4:**
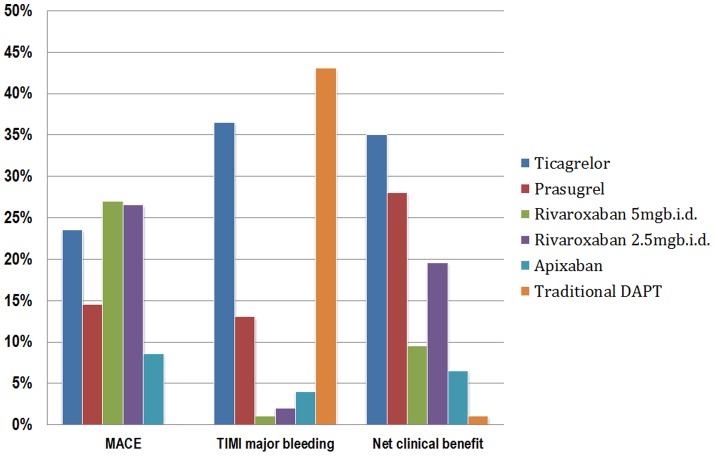
The cumulative probabilities of being among the two most efficacious regimes in reducing MACE, increasing TIMI major bleeding events and net clinical benefit. MACE: major adverse cardiac event. TIMI: thrombolysis in myocardial infarction.

The distribution of probabilities of each treatment being ranked at each of the possible six positions was presented in Table S3. The cumulative probabilities of being among the two most efficacious regimes in reducing MACE were: 27.0% for rivaroxaban 5 mg twice daily, 26.5% for rivaroxaban 2.5 mg twice daily, 23.5% for ticagrelor, 14.5% for prasugrel, 8.5% for apixaban, and0% for traditional DAPT. The cumulative probabilities of reducing TIMI major bleeding were: 43.0% for traditional DAPT, 36.5% for ticagrelor, 13% for prasugrel, 4.0% forapixaban, 2.0% for rivaroxaban 2.5 mg twice daily, and 1.0% for rivaroxaban 5 mg twice daily. The cumulative probabilities of net benefit were: 35.0% for ticagrelor, 28.0% for prasugrel, 19.5% for rivaroxaban 2.5 mg twice daily, 9.5% for rivaroxaban 5 mg twice daily, 6.5% for apixaban, and 1.0% for traditional DAPT (Figure 4).

In the sensitivity analysis, we included all the phase 2 trials investigating these antithrombotic regimes in patients with ACS. DISPERSE-2 (ticagrelor) [17], APPRAISE (apixaban) [18], and ATLAS ACS-TIMI46 (rivaroxaban) [19] were included in the sensitivity analysis, while JUMBO-TIMI 26 [20] was excluded, which investigated prasugrel both in ACS and stable coronary disease. The cumulative probabilities of the two most efficacious regimes in net benefit were similar with the result above: 35.0% for ticagrelor, 30.0% for prasugrel, 21.0% for rivaroxaban 2.5 mg twice daily, 8.0% for rivaroxaban 5 mg twice daily, 5.0% for apixaban, and 1.5% for traditional DAPT. In addition, neither the Egger's nor the Begg's tests, which assessed publication bias, showed statistical significance (both p>0.10).

## Discussion

The finding of this analysis demonstrated that the use of new antithrombotic agents resulted in significant reduction in MACE with an increased risk of major bleeding. There was a significant net benefit towards new antithrombotic regimes compared with traditional DAPT. Although no statistical significance was identified, the new P2Y_12_ receptor inhibitor, ticagrelor, showed a trend toward achieving the greatest beneficial effect compared to other regimes, which gives it the highest probability of being the optimal therapy.

There were several small-scale studies directly comparing the antiplatelet effect directly of ticagrelor with prasugrel. In ACS patients with diabetes, ticagrelor presented a significantly higher platelet inhibition than prasugrel [21] and similar results were also found in ACS patients with high on-clopidogrel platelet reactivity [22]. However, other studies did not find the differences in antiplatelet effect between these new P2Y_12_ receptor inhibitors [23], [24]. A previous meta-analysis including TRITON-TIMI 38, PLATO, and DISPERSE-2 trials indicated the similar efficacy of prasugrel to ticagrelor, while ticagrelor was associated with a significantly lower risk of any major bleeding. Of note, this meta-analysis did not include the result from TRILOGY ACS. To our knowledge, there is no RCTs comparing the new P2Y_12_ receptor inhibitors (e.g. prasugrel or ticagrelor) with novel oral anticoagulants (rivaroxaban or apixaban). It was required that large-scale RCTs directly comparing clinical value of these new antithrombotic agents were performed in order to achieve the sufficient power.

It has been reported that multiple medications may reduce patients' compliance and fixed-dose combination antihypertensive medication resulted in better compliance than the single agent [25], [26]. In ALTAS ACS 2-TIMI 51, premature discontinuation of antithrombotic agents occurred in more than one fourth of patients in either rivoraxaban or placebo group [16]. However, the overall rate of drug compliance was 82.8% in PLATO using two antithrombotic agents [14]. Thus, oral anticoagulants in addition to DAPT may be a crowd for patients with ACS, who have already taken multiple medications [27].

It is no doubt that these new antithrombotic regimes are associated with reduced rate of recurrent ischemic events. However, more potent platelet/factor Xa inhibition increases the risk of bleeding. In the current study, we found that rivaroxaban in combination with DAPT seemed likely to be the most efficacious in reducing MACE in ACS patients. However, the clinical benefit may be significantly offset by the increase in major bleeding events. This conclusion was confirmed by the recent meta-analysis, in which the administration of novel oral anticoagulant agents did not provide the net clinical benefit compared toplacebo in ACS patients, due to dramatic increase in major bleeding events [28]. Therefore, the target of antithrombotic therapies should be to inhibit platelet function or factor Xa, which may minimize the risk of ischemic and bleeding outcomes. This optimal range may be tailored to specific populations or clinical with different ischemic and bleeding risks [29].

Several limitations of this meta-analysis deserve comment. Firstly, in order to reduce heterogeneity, we included only phase 3 studies in our meta-analysis. Nevertheless, we conducted a sensitivity analysis by combining phase 2 studies with phase 3 studies, and we found the similar results to our original findings. Secondly, there was slight difference in baseline characteristics of enrolled patients in the meta-analysis due to different eligible criteria in the enrolled studies. Thirdly, although combining the major bleeding with the ischemic endpoints into a composite endpoint (net clinical benefit) has been widely used in contemporary trials [30]–[32], it may be associated with some pitfalls, such as lack of a proven link between lower bleeding rates and lower mortality rates [33]. Additionally, since this is a study-level meta-analysis, instead of patient-level meta-analysis, it was impossible to further analyze the effect of complex clinical factors, such as gender difference or the type of ACS.

### Conclusion

New antithrombotic agents are associated with significantly reduced risk of MACE, as well as an increased risk of major bleeding, in comparison with traditional DAPT. Although no significant statistical differences were identified among these new antithrombotic regimes, there was a trend in net clinical benefit favoring the new P2Y_12_ receptor inhibitor, ticagrelor. The findings may provide a support for ticagrelor plus aspirin to be an optimal antithrombotic regimen for patients with ACS.

## Supporting Information

Figure S1 **Risk of bias assessment.**
(TIF)

Table S1 **Major inclusion and exclusion criteria of the included studies.** UA =  unstable angina; NSTEMI =  non ST elevated myocardial infarction; ACS =  acute coronary syndrome; STEMI =  ST elevation myocardial infarction; PCI =  percutaneous coronary intervention; CABG =  coronary artery bypass; NYHA =  New York Heart Association.
(DOCX)

Table S2 **Medical history and medication of included studies.** MI =  myocardial infarction; CABG =  coronary artery bypass; ACEI =  angiotensin converting enzyme inhibitors; ARB =  angiotensin II receptor blocker.
(DOCX)

Table S3 **The distribution of probabilities of each treatment being ranked at each of the possible 6 positions.** DAPT  =  dual antiplatelet therapy; TIMI  =  thrombolysis in myocardial infarction.
(DOCX)

Text S1 **Search strategy (via EMBASE.com).**
(DOC)
