# Correction: Optimal Oral Antithrombotic Regimes for Patients with Acute Coronary Syndrome: A Network Meta-Analysis

**DOI:** 10.1371/journal.pone.0100047

**Published:** 2014-06-10

**Authors:** The PLOS ONE Staff

There is an error in the corresponding author’s name for this article. The correct corresponding author is Dr. Shuyang Zhang (zhangebmg@gmail.com).
