# Supplementary figures and images for: Optimal Oral Antithrombotic Regimes for Patients with Acute Coronary Syndrome: A Network Meta-Analysis
Source: PLoS One. 2014 Mar 10;9(3):e90986. doi: 10.1371/journal.pone.0090986 (PMC3948750; doi:10.1371/journal.pone.0090986)

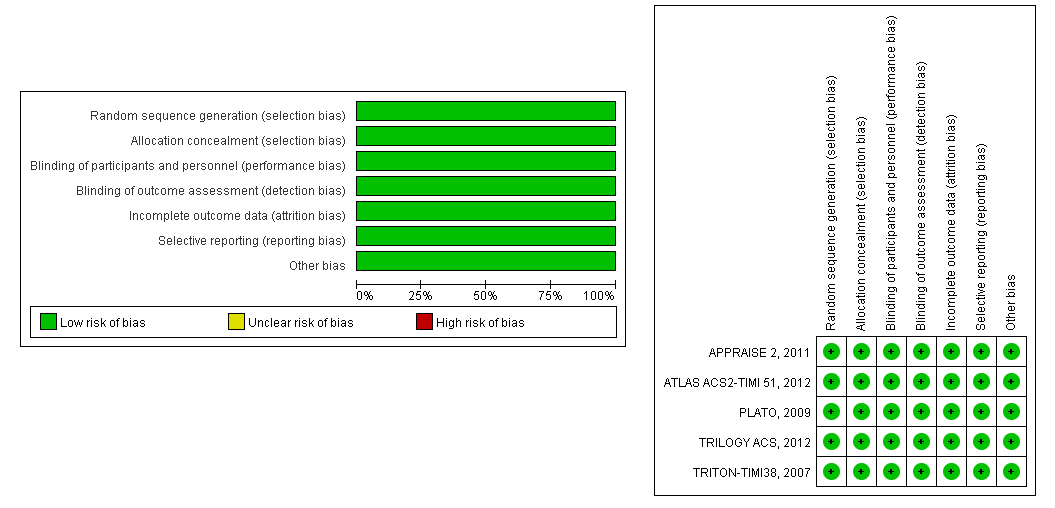

Supplement: Figure S1 — Risk of bias assessment. (TIF) [file pone.0090986.s001.tif]
