# Supplementary material for: Optimal Oral Antithrombotic Regimes for Patients with Acute Coronary Syndrome: A Network Meta-Analysis
Source: PLoS One. 2014 Mar 10;9(3):e90986. doi: 10.1371/journal.pone.0090986 (PMC3948750; doi:10.1371/journal.pone.0090986)
Supplement: Table S1 — Major inclusion and exclusion criteria of the included studies. UA = unstable angina; NSTEMI = non ST elevated myocardial infarction; ACS = acute coronary syndrome; STEMI = ST elevation myocardial infarction; PCI = percutaneous coronary intervention; CABG = coronary artery bypass; NYHA = New York Heart Association. (DOCX) [file pone.0090986.s002.docx]

| Studies | Major inclusion criteria | Major exclusion criteria |
| --- | --- | --- |
| TRITON-TIMI38 | UA/NSTEMI: ischemic symptoms lasting 10 minutes or more and occurring within 72 hours before randomization, a TIMI risk score of 3 or more, and either ST-segment deviation of 1 mm or more or elevated levels of a cardiac biomarker of necrosis.  STEMI: within 12 hours after the onset of symptoms if primary PCI was planned or within 14 days after receiving medical treatment. | Increased risk of bleeding; anemia thrombocytopenia; history of pathologic intracranial findings; use of any thienopyridine within 5 days. |
| TRILOGY ACS | 1. ACS selected for medical management without revascularization within 10 days after the index event. 2. NSTEMI: elevated cardiac markers; UA: ST-segment depression of more than 1 mm in two or more leads. 3. At least one of four risk criteria: an age of at least 60 years, the presence of diabetes mellitus, previous myocardial infarction, or previous revascularization with either PCI or CABG. | History of transient ischemic attack or stroke, PCI or CABG within the previous 30 days, renal failure requiring dialysis, and concomitant treatment with an oral anticoagulant. |
| PLATO | 1. ACS with or without ST-segment elevation, with an onset of symptoms during the previous 24 hours. 2. UA/NSTEMI: at least two of the following three criteria: ST-segment changes on electrocardiography; a positive biomarker; or one of several risk factors (age ≥60 years; previous myocardial infarction or CABG; stenosis of ≥50% in at least two vessels; previous ischemic stroke, transient ischemic attack, carotid stenosis of at least 50%, or cerebral revascularization; diabetes mellitus; peripheral arterial disease; or chronic renal dysfunction.   STEMI: persistent ST elevation of at least 0.1 mV in at least two contiguous leads or a new left bundle-branch block, and the intention to perform primary PCI. | Any contraindication against the use of clopidogrel, fibrinolytic therapy within 24 hours before randomization, a need for oral anticoagulation therapy, an increased risk of bradycardia, and concomitant therapy with a strong cytochrome P-450 3A inhibitor or inducer. |
| APPRAISE 2 | 1. ACS within the previous 7 days, with symptoms of myocardial ischemia lasting 10 minutes or more with the patient at rest plus either elevated levels of cardiac biomarkers or dynamic ST-segment depression or elevation of 0.1 mV or more. 2. Two or more of the following high-risk characteristics: an age of at least 65 years, diabetes mellitus, myocardial infarction within the previous 5 years, cerebrovascular disease, peripheral vascular disease, clinical heart failure or a left ventricular ejection fraction of less than 40% in association with the index event, impaired renal function with a calculated creatinine clearance of less than 60 ml per minute, and no revascularization after the index event. | persistent severe hypertension, severe renal dysfunction with a calculated creatinine clearance of <20 mL/min; active bleeding or a high risk for bleeding; known coagulopathy; ischemic stroke within 7 days; NYHA IV heart failure; any history of intracranial bleeding; hemoglobin < 9g/dL; platelet count< 100,000 mm3; required ongoing treatment with a parenteral or oral anticoagulant; required treatment with high dose aspirin (>325 mg daily) or a strong inhibitor of CYP3A4; a severe comorbid condition with a life expectancy of ≤6 months; acute pericarditis, active hepatobiliary disease, and women who were pregnant, breastfeeding, or of childbearing potential and unable to use an acceptable method of birth control. |
| ATLAS ACS2-TIMI 51 | ACS (Patients who were under 55 years of age had either diabetes mellitus or a previous myocardial infarction in addition to the index event.) | A platelet count of less than 90,000 per cubic millimeter, a hemoglobin level of less than 10 g per deciliter, or a creatinine clearance of less than 30 ml per minute at screening; clinically significant gastrointestinal bleeding within 12 months before randomization; previous intracranial hemorrhage; and previous ischemic stroke or transient ischemic attack in patients who were taking both aspirin and a thienopyridine. |
