# Supplementary material for: Optimal Oral Antithrombotic Regimes for Patients with Acute Coronary Syndrome: A Network Meta-Analysis
Source: PLoS One. 2014 Mar 10;9(3):e90986. doi: 10.1371/journal.pone.0090986 (PMC3948750; doi:10.1371/journal.pone.0090986)
Supplement: Table S2 — Medical history and medication of included studies. MI = myocardial infarction; CABG = coronary artery bypass; ACEI = angiotensin converting enzyme inhibitors; ARB = angiotensin II receptor blocker. (DOCX) [file pone.0090986.s003.docx]

| Studies | Hypertension | Diabetes | Hypercholesterolemia | Previous  MI | Previous  CABG | Creatinine  clearance | ACEI/ARB | Beta-blocker | Statin |
| --- | --- | --- | --- | --- | --- | --- | --- | --- | --- |
| TRITON-TIMI38 | 64 | 23 | 56 | 18 | 7.5 | / | 75.5 | 88 | 92 |
| TRILOGY ACS | 82 | 38 | 59.2 | 43.1 | 15.7 | 73 | 75.4 | 77.8 | 83.4 |
| PLATO | 65.5 | 25 | 46.7 | 20.6 | 5.9 | / | 87.8 | 89.5 | 89.5 |
| APPRAISE 2 | / | 47.9 | / | 26.2 | / | / | 79.4 | 76.7 | 83.6 |
| ATLAS ACS2-TIMI 51 | 67.4 | 32.1 | 48.7 | 26.7 | / | 85 | 38.7 | 65.9 | 83.6 |
