# Supplementary material for: Optimal Oral Antithrombotic Regimes for Patients with Acute Coronary Syndrome: A Network Meta-Analysis
Source: PLoS One. 2014 Mar 10;9(3):e90986. doi: 10.1371/journal.pone.0090986 (PMC3948750; doi:10.1371/journal.pone.0090986)
Supplement: Table S3 — The distribution of probabilities of each treatment being ranked at each of the possible 6 positions. DAPT = dual antiplatelet therapy; TIMI = thrombolysis in myocardial infarction. (DOCX) [file pone.0090986.s004.docx]

| **Drugs** | **Major adverse cardiac events** | | | | | | **TIMI major bleeding** | | | | | | **Net clinical benefit** | | | | | |
| --- | --- | --- | --- | --- | --- | --- | --- | --- | --- | --- | --- | --- | --- | --- | --- | --- | --- | --- |
|  | **Rank 1** | **Rank 2** | **Rank 3** | **Rank 4** | **Rank 5** | **Rank 6** | **Rank 1** | **Rank 2** | **Rank 3** | **Rank 4** | **Rank 5** | **Rank 6** | **Rank 1** | **Rank 2** | **Rank 3** | **Rank 4** | **Rank 5** | **Rank 6** |
| Traditional DAPT | 0.50 | 0.37 | 0.11 | 0.03 | 0.00 | 0.00 | 0.00 | 0.01 | 0.02 | 0.12 | 0.44 | 0.42 | 0.12 | 0.37 | 0.32 | 0.16 | 0.02 | 0.00 |
| Apixaban | 0.30 | 0.25 | 0.16 | 0.12 | 0.09 | 0.08 | 0.17 | 0.17 | 0.50 | 0.08 | 0.04 | 0.04 | 0.50 | 0.16 | 0.12 | 0.10 | 0.07 | 0.06 |
| Prasugrel | 0.04 | 0.14 | 0.29 | 0.24 | 0.18 | 0.11 | 0.02 | 0.04 | 0.11 | 0.57 | 0.16 | 0.10 | 0.02 | 0.06 | 0.13 | 0.22 | 0.33 | 0.23 |
| Rivaroxaban 2.5mgb.i.d. | 0.05 | 0.08 | 0.14 | 0.20 | 0.26 | 0.27 | 0.20 | 0.49 | 0.23 | 0.05 | 0.02 | 0.02 | 0.09 | 0.15 | 0.16 | 0.21 | 0.19 | 0.20 |
| Rivaroxaban 5mgb.i.d. | 0.05 | 0.08 | 0.14 | 0.20 | 0.26 | 0.28 | 0.59 | 0.27 | 0.09 | 0.03 | 0.01 | 0.01 | 0.24 | 0.22 | 0.18 | 0.16 | 0.12 | 0.07 |
| Ticagrelor | 0.06 | 0.09 | 0.17 | 0.21 | 0.21 | 0.26 | 0.02 | 0.03 | 0.06 | 0.16 | 0.32 | 0.41 | 0.02 | 0.04 | 0.08 | 0.16 | 0.26 | 0.44 |
