# Supplementary material for: Optimal Oral Antithrombotic Regimes for Patients with Acute Coronary Syndrome: A Network Meta-Analysis
Source: PLoS One. 2014 Mar 10;9(3):e90986. doi: 10.1371/journal.pone.0090986 (PMC3948750; doi:10.1371/journal.pone.0090986)
Supplement: Text S1 — Search strategy (via EMBASE.com). (DOC) [file pone.0090986.s005.doc]

1. ‘antithrombin’/exp OR ‘antithrombin’
2. ‘factorxa’/exp OR ‘factor xa’
3. ‘factorxa inhibitor’/exp OR ‘factor xa inhibitor’
4. ‘ximelagatran’/exp OR ‘ximelagatran’
5. ‘dabigatran’/exp OR ‘dabigatran’
6. ‘apixaban’/exp OR ‘apixaban’
7. ‘rivaroxaban’/exp OR ‘rivaroxaban’
8. ‘darexaban’/exp OR ‘darexaban’
9. ‘prasugrel’/exp OR ‘prasugrel’
10. ‘ticagrelor’/exp OR ‘ticagrelor’
11. ‘acute coronary syndrome’/exp OR ‘acute coronary syndrome’
12. ‘myocardial infarction’/exp OR ‘myocardial infarction’\
13. #1 OR #2 OR #3 OR #4 OR #5 OR #6 OR #7 OR #8 OR #9 OR #10
14. #11 OR #12
15. #13AND #14
16. #15 AND ‘randomized controlled trial’/de
